# Optimized protocol for whole-mount RNA fluorescent *in situ* hybridization using oxidation-mediated autofluorescence reduction on mouse embryos

**DOI:** 10.1016/j.xpro.2023.102603

**Published:** 2023-09-23

**Authors:** Angela Morabito, Jonas Malkmus, Anna Pancho, Aimée Zuniga, Rolf Zeller, Rushikesh Sheth

**Affiliations:** 1 Developmental Genetics, Department of Biomedicine, University of Basel, 4058 Basel, Switzerland

**Keywords:** Developmental Biology, Microscopy, Gene Expression, Antibody, *In situ* Hybridization

## Abstract

Tissue autofluorescence poses significant challenges for RNA and protein analysis using fluorescence-based techniques. Here, we present a protocol that combines oxidation-mediated autofluorescence reduction with detergent-based tissue permeabilization for whole-mount RNA-fluorescence *in situ* hybridization (FISH) on mouse embryonic limb buds. We describe the steps for embryo collection, fixation, photochemical bleaching, permeabilization, and RNA-FISH, followed by optical clearing of RNA-FISH and immunofluorescence samples for imaging. The protocol alleviates the need for digital image post-processing to remove autofluorescence and is applicable to other tissues, organs, and vertebrate embryos.

## Highlights

• Maximal suppression of autofluorescence using OMAR photochemical bleaching
• The technique is suitable for whole-mount RNA-FISH and immunofluorescence
• From embryo collection to 2D and 3D image analysis in a week

## Abstract (editor highlights)

Publisher’s note: Undertaking any experimental protocol requires adherence to local institutional guidelines for laboratory safety and ethics.

## Graphical abstract

**Figure:**
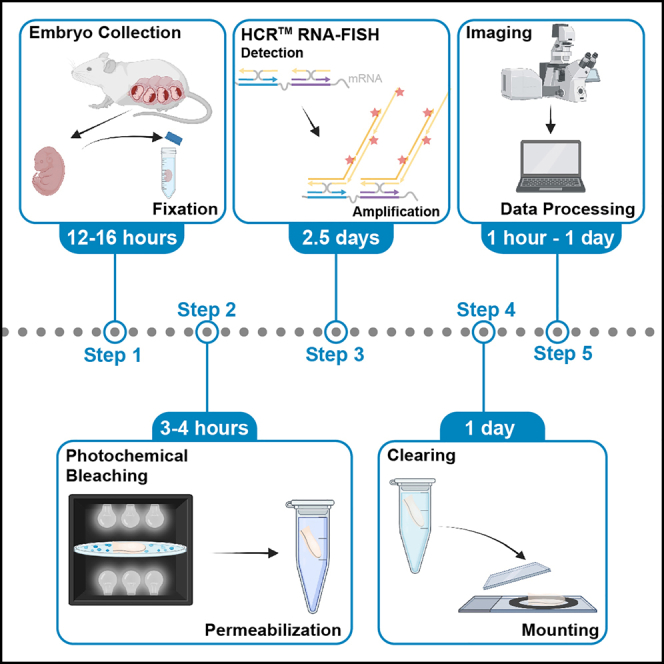

## Before you begin

Tissue autofluorescence emanating from various sources poses a complex challenge for high-sensitivity detection of fluorescently labelled RNA probes and antibody staining.^1^ Although these problems can -in some cases- be ameliorated by post-processing of raw images.^2^ It is preferable to eliminate tissue autofluorescence at the source and prior to fluorescent labelling. Therefore, various pre-treatments such as irradiation-by-light or chemicals are used to eliminate autofluorescence on tissue sections.^3^^,^^4^^,^^5^^,^^6^^,^^7^ At the same time, this has remained challenging for fluorescence detection on whole mounts such as embryos, tissues, and organs. Here, we describe the adaptation of a photochemical pre-treatment^8^ suited for fluorescent whole-mount analysis that consistently reduces and most often eliminates tissue and blood vessel autofluorescence. The OMAR treatment improves the signal-to-noise ratio for whole-mount Hybridization Chain Reaction (HCR) RNA fluorescent *in situ* hybridization (RNA-FISH). This study also shows that OMAR can be used for whole-mount immunofluorescence analysis.

**Figure 1:**
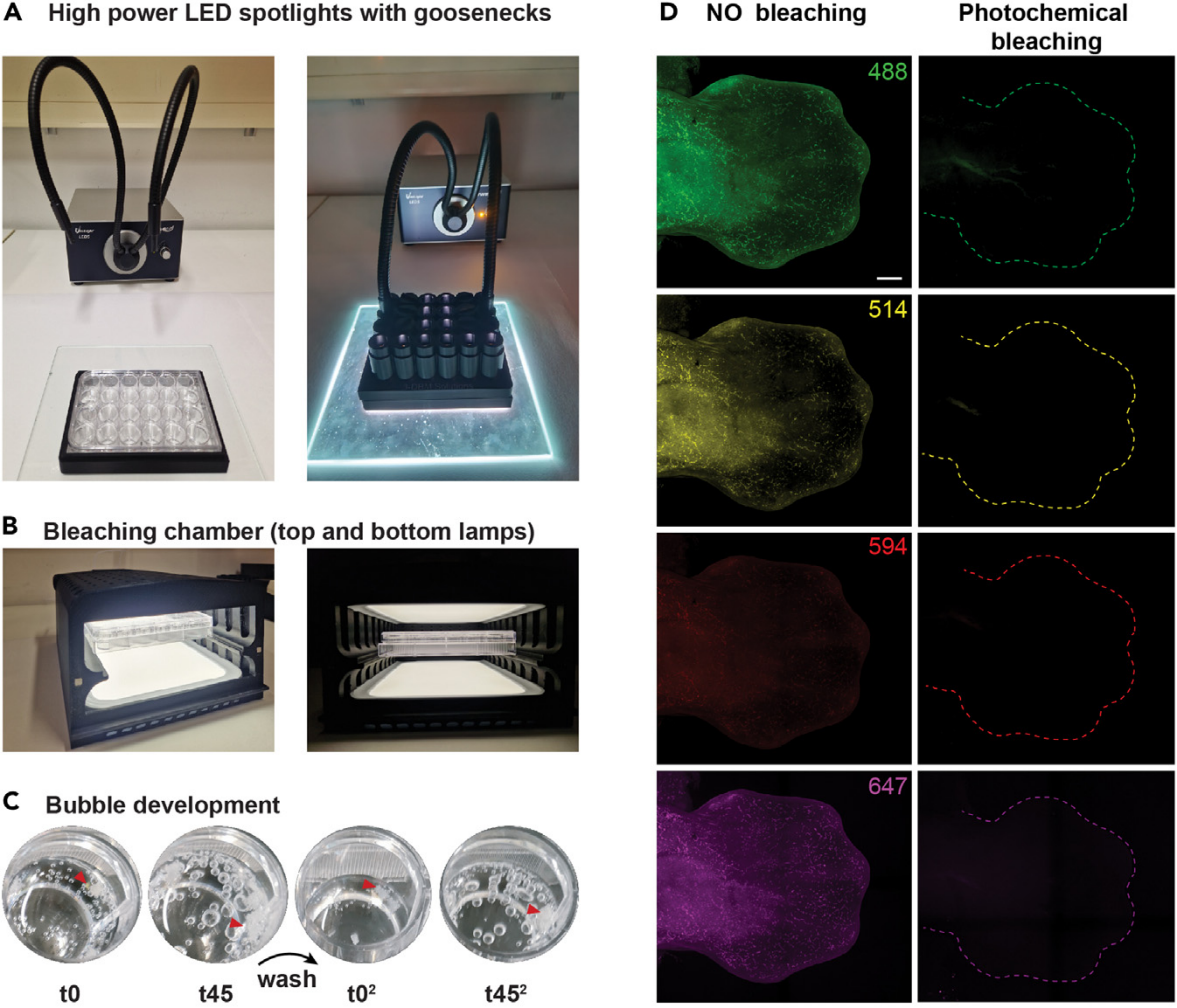
OMAR photochemical bleaching of embryonic tissue Photochemical bleaching can be performed using intense cold-white LED lights in two ways. (A) Using LED spotlight light with gooseneck glass fiber transmission (VWR VisiLight LED8, goosenecks provide a 1600 lumen spotlight). (B) Alternatively, use daylight lamp pads for Light therapy (homogenous LED lighting with 20000 lumen). The cases in panels A and B are homemade to provide the necessary spacing, eye protection from light reflection, and optimal heat management. Details for 3D printing the cases are available on request. (C) Bubble formation during the 2 × 45 min OMAR photochemical bleaching. (D) Side-by-side comparison of autofluorescence in four confocal channels (488, 514, 594, and 647 nm) showing the contralateral forelimb buds embryo without (left) and with OMAR photochemical bleaching (right), respectively. Scale bar: 200 μm.

Briefly, OMAR requires a high-intensity cold white light source. For example, high-power LED spotlights on flexible goosenecks (Figure 1A) or two LED daylight panels (20000 lumen) can be used (Figure 1B). As autofluorescence varies significantly between tissues, the efficacy of the available LED light source(s) in eliminating autofluorescence must be established in a test series. Use the fixed tissues or organs of interest to carry out steps 1–10 of the step-by-step protocol, followed by mounting and imaging the samples. Successful oxidation reaction manifests itself by the appearance of an increasing number and size of bubbles in the solution and around the sample during the two photochemical pre-treatments (Figure 1C). The LED light source is appropriate if the endogenous autofluorescence is low or absent in all channels of interest (Figure 1D, left panels: untreated controls, right panels: OMAR treated mouse limb buds). The OMAR photochemical autofluorescence reduction protocol is used in combination with the HCR RNA-FISH (v3.0) approach from Molecular Instruments, and all essential information is available on the molecular instrument website.^9^ It is advisable to first test the probe(s) of interest with the respective amplifiers in a small experiment.

### Institutional permissions

All experiments conducted with *Mus musculus* embryos of both sexes at the relevant developmental stages are performed respecting the Swiss laws governing animal research and the mandatory 3R principles. HCR RNA-FISH is in line with the 3R reduce and refine principles as it allows the detection of several different transcripts per sample, which reduces the number of mouse embryos needed for analysis. Our animal research using mouse embryos was evaluated and approved by the Regional Commission on Animal Experimentation of the Veterinary Office of both cantons of Basel.

## Key resources table

**Table:** 

REAGENT or RESOURCE	SOURCE	IDENTIFIER
**Antibodies**		

Phospho-SMAD1 (Ser463/465)/SMAD5 (Ser463/465)/SMAD9 (Ser465/467) (D5B10) rabbit (1:200 dilution)	Cell Signaling Technology	CAT 13820S
Sox9 (1:200 dilution)	Merck Millipore	CAT AB5535
Anti-phospho-histone H3 (Ser10) (1:200 dilution)	Sigma-Aldrich	CAT 06-570
Donkey anti-rabbit IgG (H + L) highly cross-adsorbed secondary antibody, Alexa Fluor 647 (1:1000 dilution)	Thermo Fisher Scientific	CAT A-31573
Donkey anti-rabbit IgG (H + L) highly cross-adsorbed secondary antibody, Alexa Fluor 555 (1:1000 dilution)	Thermo Fisher Scientific	CAT A-31572

**Chemicals, peptides, and recombinant proteins**		

Paraformaldehyde	Sigma-Aldrich	SKU P6148-1Kg
Methanol	VWR	CAT 20903.368
Tween 20	Sigma-Aldrich	SKU 93773-1kg
Hydrogen peroxide 33% w/v	AppliChem	SKU 141077
Sodium hydroxide	Sigma-Aldrich	SKU 1.06498.1000
Sodium dodecyl sulfate	Sigma-Aldrich	SKU L3771-500g
Tris ultrapure	AppliChem	SKU A1086,1000
EDTA disodium salt 2-hydrate for molecular biology	AppliChem	SKU A2937,1000
Sodium chloride	AppliChem	SKU A2942,1000
Sodium citrate tribasic dihydrate	Sigma-Aldrich	SKU C8532-500
D-(-)-fructose	Sigma-Aldrich	SKU F0127-500g
Glycerol	AppliChem	SKU 131339.1211
Triton X-100	Sigma-Aldrich	SKU T8787-100ml
Bovine serum albumin	Sigma-Aldrich	SKU A2153-100g
Donkey serum	Sigma-Aldrich	SKU S30-100ml
DAPI	Sigma-Aldrich	SKU D9542-1mg
Probe hybridization buffer (HCR)	Molecular Instruments	N/A
Probe wash buffer	Molecular Instruments	N/A
Amplification buffer	Molecular Instruments	N/A

**Experimental models: Organisms/strains**		

Embryos: Swiss albino mouse	Janvier Labs	Wild type (male and female embryos of distinct stages)

**Oligonucleotides**		

Mouse *Hand2* HCR v3.0 probe set (B2) set size: 20	Molecular Instruments	GenBank: NM_010402.4
Mouse *Gli3* HCR v3.0 probe set (B4) set size: 20	Molecular Instruments	GenBank: NM_008130
Mouse *Shh* HCR v3.0 probe set (B1) set size: 20	Molecular Instruments	GenBank: NM_009170
Mouse *Grem1* HCR v3.0 probe set (B2) set size: 20	Molecular Instruments	GenBank: NM_011824.4
Mouse *Fgf4* HCR v3.0 probe set (B2) set size: 20	Molecular Instruments	GenBank: NM_010202.6
Mouse *Cyp26b1* HCR v3.0 probe set (B4) set size: 20	Molecular Instruments	GenBank: NM_175475.3
Mouse *Meis2* HCR v3.0 probe set (B5) set size: 20	Molecular Instruments	GenBank: NM_001159568.1
Mouse *Sox9* HCR v3.0 probe set (B1) set size: 20	Molecular Instruments	GenBank: NM_011448.4
Mouse *Bmp2* HCR v3.0 probe set (B5) set size: 20	Molecular Instruments	GenBank: NM_007553.3
Mouse *Msx1* HCR v3.0 probe set (B3) set size: 20	Molecular Instruments	GenBank: NM_010835.2
Mouse *Noggin* HCR v3.0 probe set (B4) set size: 20	Molecular Instruments	GenBank: NM_008711.2
Mouse *Col2a1* HCR v3.0 probe set (B1) set size: 20	Molecular Instruments	GenBank: NM_031163
Mouse *Gdf5* HCR v3.0 probe set (B2) set size: 20	Molecular Instruments	GenBank: NM_008109.4
B1 514 HCR amplifier	Molecular Instruments	N/A
B2 514 HCR amplifier	Molecular Instruments	N/A
B5 514 HCR amplifier	Molecular Instruments	N/A
B1 594 HCR amplifier	Molecular Instruments	N/A
B2 594 HCR amplifier	Molecular Instruments	N/A
B4 594 HCR amplifier	Molecular Instruments	N/A
B2 647 HCR amplifier	Molecular Instruments	N/A
B3 647 HCR amplifier	Molecular Instruments	N/A
B4 647 HCR amplifier	Molecular Instruments	N/A

**Software and algorithms**		

ImageJ2 version 2.9.0/1.53t (Fiji2)	National Institutes of Health (open source)	http://imagej.net/Fiji
Imaris 9.90	Oxford Instruments	https://imaris.oxinst.com/packages
VisiView Premier Image Acquisition Software	Visitron Systems Microscopy and Imaging	N/A
ZEN3.1 Black LS	Zeiss	N/A

**Other**		

Dissection microscope	Leica Microsystems	M60
Glass vial	Roth	LC84.1
Forceps	Dumont Swiss made	0208-5/45-PO
Microtubes 2 mL	Life System Design	4ti.0798
Nutator	Fisher Scientific	10470655
ThermoMixer F1.5	Eppendorf	EP5384000012
Light therapy LED lamp – 20000 lumen – warm white light	My Sun Market	Model: XZ1902
24-well plates	Costar	3738
iSpacer circular well	Sunjin Lab	IS312-IS317
Coverslips round	VWR	631-0153
SuperFrost Plus adhesion slides	Fisher Scientific	15438060
Magnetic stirrers	Fisher Scientific	17722539
Tissue-Tek cryomolds	Biosystems	81-0791-00
Transfer pipettes, 3 mL	LLG Lab Logistics	4.672 671
Microwave	Menumaster	N/A
Digital handheld refractometer	IGZ Instruments AG	DR201-95
Visitron spinning disk confocal	Nikon Ti-E	N/A
Zeiss Lightsheet 7 microscope	Zeiss	2583000113 INDIMO:2931

## Materials and equipment

### Preparation of fixative

4% paraformaldehyde (PFA): add 1 g of PFA powder per 25 mL of PBS (pH 7.4).

***Note:*** Heat the solution to 50°C–60°C in a chemical hood until the PFA powder is dissolved.

**CRITICAL:** Paraformaldehyde is toxic: a potential carcinogen and irritant. Wear gloves and handle with care using a laminar flow chemical hood. Dispose formaldehyde waste into a dedicated chemical waste container in the chemical hood following institutional guidelines for safe removal and final disposal.

### Methanol dehydration/rehydration series

• 25% Methanol solution: add 50 mL Methanol (100%) in 150 mL PBST.
• 50% Methanol solution: add 100 mL Methanol (100%) in 100 mL PBST.
• 75% Methanol solution: add 150 mL Methanol (100%) in 50 mL PBST.
• 100% Methanol.

**CRITICAL:** Exposure to significant quantities of Methanol can cause eye damage (blindness). Handle only small quantities with gloves. The methanol series can be stored in a ventilated safety cabinet for 3 months at room temperature (24°C). Long-term storage of Methanol requires fully sealed containers with no evaporation.

**Table:** Bleaching Solution

Reagent	Final concentration	Amount
PBS 10× (pH 7.4)	1×	3 mL
H_2_O_2_ 33%	5%	4.5 mL
NaOH (1 M)	27 mM	0.8 mL
ddH_2_O	N/A	22 mL
**Total**	**N/A**	**30.3 mL**

**Table:** Detergent Solution

Reagent	Final concentration	Amount
10% SDS	4%	40 mL
10% Tween 20	0.5%	5 mL
Tris HCl (1 M) pH 7.4	50 mM	5 mL
EDTA (0.5 M) pH 8	1 mM	0.2 mL
NaCl (5 M)	150 mM	3 mL
ddH_2_O	N/A	46.8 mL
**Total**	**N/A**	**100 mL**

**Table:** PBST

Reagent	Final concentration	Amount
PBS 10× (pH 7.4)	1×	5 mL
10% Tween-20	0.1%	0.5 mL
ddH_2_O	N/A	44.5 mL
**Total**	**N/A**	**50 mL**

**Table:** 5× SSCT

Reagent	Final concentration	Amount
20× Sodium chloride Sodium citrate (SSC) pH 7.4	5×	10 mL
10% Tween 20	0.1%	0.4 mL
ddH_2_O	N/A	29.6 mL
**Total**	**N/A**	**40 mL**

### DAPI (1000× stock)

1 mg/mL DAPI (Sigma-Aldrich D9542) in H_2_O (pH 7.4)

**Table:** Blocking Solution

Reagent	Final concentration	Amount
10% Triton X-100	0.4%	2 mL
10% BSA	1%	5 mL
10% Tween 20	0.1%	0.5 mL
Donkey serum	10%	5 mL
10× PBS (pH 7.4)	1×	5 mL
ddH_2_O	N/A	32.5 mL
**Total**	**N/A**	**50 mL**

### Wash solution

• 0.4% Triton X-100: add 0.4 mL of Triton X-100 (10%) in 10 mL of PBS (pH 7.4).
• 0.1% Triton X-100: add 0.1 mL of Triton X-100 (10%) in 10 mL of PBS (pH 7.4).

**Table:** Fructose-Glycerol-Clearing Solution

Reagent	Final concentration	Amount
D-Fructose (solid)	2.5 M	29.7 g
Glycerol	60% (vol/vol)	33 mL
Tris pH 7.4 (1 M)	10 mM	7 mL
**Total**	**N/A**	**66 mL**

***Note:*** Dissolve the D-Fructose in 10 mM Tris pH 7.4. Heat in the microwave without boiling (5–10 s pulses with mixing in between). When the fructose is dissolved, stir the solution for 10 min to cool about 30°C –35°C before adding the glycerol. The solution must be homogenous without bubbles. Prepare fresh for optimal results.

## Step-by-step method details

### Embryo collection, fixation, and dehydration

**Timing: ∼24 h**

This part describes the collection of mouse embryonic samples for whole-mount HCR RNA-FISH. Samples are fixed in 4% formaldehyde prepared in PBS to preserve tissue integrity and allow long-term storage in 100% Methanol.

***Note:*** The fixation time depends on the size and age of the embryos or other biological samples of interest. Suppose optimal fixation times for a given tissue are not known. In that case, empirically determine the time in a test experiment. The following protocol works well for mouse embryos fixed for 12–16 h ranging from embryonic days E9.5 to E13.5.

1. Euthanize mice and collect embryos according to the institutional ethical committee guidelines.
  a. Dissect and wash embryos in ice-cold PBS buffer to remove as much blood as possible.

### Fixation and dehydration

2. Transfer freshly dissected embryos to a glass vial.
  a. Add minimally 5 mL of ice-cold 4% PFA in 1x PBS (pH 7.4).
  b. Place the glass vial on a rotating or gently rocking platform for 12–16 h at 4°C to fix the embryos.
3. Remove the fixative solution carefully.
  a. Wash with 5 mL 1x PBS minimally 3 times for 5 min.
  b. Wash 2 times 5 min in PBST.

***Note:*** Store PBST at room temperature (24°C).

4. Remove PBST and dehydrate the embryos in the Methanol (MeOH) dehydration series for 10 min each at room temperature (24°C) on a rocking platform. Using 5 mL per tube for each step:
  a. 25% MeOH / 75% PBST.
  b. 50% MeOH / 50% PBST.
  c. 75% MeOH / 25% PBST.
  d. 100% MeOH.
  e. 100% MeOH.

**CRITICAL:** Both PFA and Methanol are hazardous toxic substances. Therefore, work under a laminar flow chemical hood and strictly follow institutional safety and correct waste disposal guidelines.

**Pause point:** Keep dehydrated embryos in 100% Methanol at -20°C for long-term storage.

***Note:*** The following whole-mount HCR RNA-FISH protocol works well with embryos fixed with 4% PFA for 12–16 h at 4°C and then stored in 100% methanol at -20°C up to a year.

5. Remove the 100% Methanol and rehydrate the embryos in the methanol rehydration series for 10 min each at room temperature (24°C) using a rocking platform. Using minimally 5 mL per tube for each step:
  a. 75% MeOH / 25% PBST.
  b. 50% MeOH / 50% PBST.
  c. 25% MeOH / 75% PBST.
  d. 100% PBST.
  e. 100% PBST.

***Note:*** Proper embryo dehydration and rehydration are essential before proceeding to the next step.

6. If necessary, dissect the embryonic tissues, such as limb buds.
  a. Transfer the dissected tissue in PBST into individual wells of a 24-well tissue culture plate.
  b. Wash two times for 5 min in 1 mL PBS.

***Note:*** For limb buds, we remove as much trunk tissue as possible, which allows for a minimal volume of the HCR probe and amplifier and facilitates the mounting of samples for imaging. Alternatively, increase the volumes of all solutions and the probe and amplifier concentrations as required.

### Oxidation-mediated autofluorescence reduction (OMAR)

**Timing: ∼2 h 30 min**

In this step, OMAR photochemical bleaching of mouse embryonic limb buds eliminates or significantly reduces tissue and blood vessel autofluorescence in mouse limb buds prior to proceeding with whole-mount HCR RNA-FISH.

***Note:*** Hydrogen peroxide is the most widely used pigment decoloring/bleaching agent, known to degrade the pigment (such as heme, lipofuscin, melanin, etc.) and the protein structure. Also, strong acids (e.g., acetone) and strong alkalis (e.g., NaOH) can dissociate heme structure, allowing efficient pigment bleaching. Photobleaching with strong UV or white light is routinely used on tissue sections. However, hydrogen peroxide, sodium hydroxide, or exposure to LED light alone is inefficient in obtaining the desired outcome on whole-mount tissue. The OMAR is based on light and base-catalyzed oxidation.^8^ The limb buds are immersed in the bleaching solution containing hydrogen peroxide and sodium hydroxide and exposed to an LED spotlight source (Figure 1A) or LED light plates (20000 lumen, Figure 1B). Hydrogen peroxide combined with sodium hydroxide generates sodium peroxide and thus creates a strong oxidative environment. While exposition to the LED light not only excites the endogenous fluorescent molecules, it also ionizes biomolecules, generating intermediate reactive oxidation species (ROS).^10^ The combined effect of H_2_O_2_ and NaOH in the solution and LED light improves tissue permeability. Additionally, ROS generated in the process oxidizes the heme and other biomolecules, which helps quench the background fluorescence across the visible spectrum.

**CRITICAL:** A successful photo-oxidation reaction manifests itself in microbubbles that grow in number and size during light exposure (Figure 1C). It is important to note that the specific reaction and outcome may vary depending on factors such as the duration of exposure and the intensity and wavelength of LED lights and properties of tissue and organ. Overexposure may damage the tissue and cellular components; therefore, carefully optimize the reaction time and the number of cycles beforehand for the tissue of interest.

**CRITICAL:** The bleaching solution must be prepared fresh before use.

7. Remove the PBS and add 1 mL Bleaching Solution.
  a. Expose the plate to intense LED light for 45 min.

***Note:*** Heat can damage the tissue and lead to undesirable results. Therefore, keep an approximately 2 cm–3 cm distance between the plate and light source to avoid sample heating. Avoid excessive space between the light source and samples, as it may slow the photochemical reaction (see troubleshooting, problem 1).

8. Remove the Bleaching Solution.
  a. Wash the embryos twice with 1 mL PBS for 5 min at room temperature (24°C) on a rocking platform (20 oscillations/min).

**CRITICAL:** Carefully remove the Bleaching Solution and wash it with PBS. Gently add the PBS to avoid damaging the tissue. Also, be aware that the tissues, such as limb buds, may stick to the plastic wells (see troubleshooting, problem 2).

9. Remove the PBS and add 1 mL of freshly prepared Bleaching Solution.
  a. Expose the microwell plate to LED light for another 45 min.

***Note:*** In our hands, two rounds of photochemical bleaching are sufficient to reduce or eliminate autofluorescence on mouse embryonic limb buds. To find a suitable condition for embryonic tissue or organ of interest, we recommend that users perform a pilot photochemical bleaching experiment with at least three conditions (see troubleshooting, problem 3): 2 times 30 min, 2 times 45 min, and 4 times 30 min. One could also test 2 or 3 times 15 min for fragile tissues or smaller samples.

10. Remove the Bleaching Solution and wash the limb buds/tissue samples at least three times with 1 mL PBST for 10 min each at room temperature (24°C) on a rocking platform.

**CRITICAL:** Check the limb buds/tissue samples under the microscope for any remaining bubbles. If bubbles persist, wash the samples longer in PBST with gentle shaking (300 rpm; see troubleshooting, problem 4).

### HCR RNA-FISH protocol

**Timing: ∼2.5 days**

This step describes the modified whole-mount HCR RNA-FISH protocol to circumvent the use of proteinase-K and the necessary post-fixation step, as this may affect tissue morphology and cause fixation-related autofluorescence.

***Note:*** The proteinase-K-based permeabilization is substituted by detergent-based tissue permeabilization that does not require post-fixation.^11^ In contrast to Bruce et al.,^11^ the SDS concentration is increased to 4% for mouse limb buds. The volume of solutions, probe concentration, hairpin volume, and incubation times are optimized for 4–6 mouse limb buds per tube (see troubleshooting section, problem 5).

***Note:*** Store the detergent solution in the dark at room temperature (24°C). Maximally 1 month.

11. Transfer the limb buds in 1 mL PBST to a clean 2 mL microcentrifuge tube.
  a. Carefully remove all PBST.
  b. Replace with 1 mL Detergent Solution, and incubate for 2 h at 37°C on a shaker at 300 rpm.
12. Remove the Detergent Solution.
  a. Wash the limb buds twice with 1 mL PBST for 5 min at room temperature (24°C).

***Note:*** For samples smaller than mouse limb buds at E10.5 and/or fragile epithelial tissues, consider reducing the SDS concentration to, e.g., 1% and/or the incubation time to 30 min or 1 h. Empirically determine the optimal SDS concentration and incubation time beforehand. Note that tissue permeabilization using an SDS-containing detergent solution also helps delipidation and facilitates optical clearing (step 25).

13. Remove the PBST.
  a. Equilibrate the limb buds in 100 μL of Probe Hybridization buffer (Molecular Instruments) for 10 min at 24°C on a shaker at 300 rpm.
14. Discard the Probe Hybridization buffer.
  a. Replace it with 200 μL of pre-warmed (37°C) Probe Hybridization buffer for 1 h or longer at 37°C, 300 rpm.

***Note:*** The probe hybridization buffer volume depends on limb bud size and numbers per tube.

**Pause point:** At this step, limb buds can be stored in Probe Hybridization buffer at -20°C for several months.

15. Meanwhile, prepare the desired HCR probe mixes by adding 1 μL of each probe set (1 μM stock) to 100 μL of pre-warmed (37°C) Probe Hybridization buffer.
16. Remove the pre-hybridization solution.
  a. Replace it with the prepared probe mixes (100 μL per tube).
  b. Incubate for 12–16 h at 37°C on a shaking platform set to 300 rpm at 37°C.
17. Remove the probe mixes.
  a. Add 200 μL of pre-warmed Probe Wash buffer (Molecular Instruments).
  b. Perform four washes with a pre-warmed Probe Wash buffer of 20 min each at 37°C with shaking at 300 rpm.

***Note:*** Recover and store the used probe mixes at -20°C to reuse. We have successfully reused probes at least two to three times.

18. Remove the Probe Wash buffer.
  a. Wash three times with 1 mL 5x SSCT (pH 7.4) for 20 min per wash at room temperature (24°C).

***Note:*** Store 5× SSCT at room temperature (24°C).

19. Remove the 5x SSCT.
  a. Add 200 μL of pre-equilibrated Amplification buffer (Molecular Instruments) for 5 min at 24°C.

**CRITICAL:** Pre-equilibrate the Amplification buffer at 24°C before use.

20. Replace the Amplification buffer with 200 μL of fresh pre-equilibrated Amplification buffer.
  a. Pre-amplify the limb bud samples for at least 1 h or more at 24°C.

***Note:*** Meanwhile, prepare the hairpins by heating H1 and H2 separately in separate tubes for 90 s at 95°C. Let them cool down to room temperature (24°C) for 30 min in a dark place. Mix hairpin H1 and H2 in the Amplification buffer. Per tube (containing 4–6 limb buds), use 2 μL of Hairpin H1 and H2 (3 μM stock) per fluorophore in 100 μL Amplification buffer. Protect the tubes from light.

**CRITICAL:** From this step onward, protect the samples in their tubes from light during all steps**.**

21. Replace the Amplification buffer with 100 μL hairpin mix per tube.
  a. Incubate the limb buds for 12–16 h on a shaker (300 rpm) at 24°C. Protect the tubes from light.
22. Recover the hairpin mixes.
  a. Wash the limb buds three times 5 min with 1 mL 5x SSCT at room temperature (24°C) on a rocking platform (20 oscillations/min). Protect the tubes from light.

***Note:*** Do not discard the hairpin mixes; we have successfully reused hairpin mixes at least two to three times. Store in the dark at 4°C (for immediate reuse) or −20°C for long-term storage.

23. Transfer the limb bud samples to a clean microcentrifuge tube. Protect the samples from light.
  a. Wash the samples 6 times for 30 min each by incubating them in 1 mL 5x SSCT containing DAPI (diluted 1:1000 from the 1 mg/mL stock) on a rocking platform.

***Note:*** Store DAPI 1 mg/mL stock at -20°C in dark. DAPI staining is recommended as it helps to orient the samples during the mounting procedure (steps 26–28) for fluorescence microscopy. If DAPI is not required, wash samples 6 times with 5x SSCT.

24. Transfer the limb buds to a new tube protected from light.
  a. Wash 3 times for 5 min with 1 mL 5x SSCT (without DAPI).
  b. The samples are ready for optical clearing and imaging.

**Pause point:** If required, store the samples in the dark in 5X SSCT solution for several days.

### Optical clearing and sample preparation for imaging

**Timing: ∼24 h**

This step describes the optical clearing preparation of samples for confocal and/or light sheet microscopy. The samples prepared using this protocol do not require further delipidation and depigmentation. A water-based, non-toxic fructose and glycerol clearing method is used for optical clearing and refractive index matching.^12^

25. Transfer limb buds to a new multi-well plate. Protect the plate from light.
  a. Add freshly prepared Fructose-Glycerol-Clearing solution.
  b. Let the tissue clear for 24 h at room temperature (24°C) on a rocking platform (20 oscillations/min).

**CRITICAL:** Maintaining a pH of 7.4 is essential for the stability of fluorophores and the long-term storage of HCR RNA-FISH samples. Changes in pH may impact the intensity or cause a complete loss of fluorescence signals.

***Note:* ∼**24 h of clearing is sufficient for most embryonic samples of the size of limb buds up to E12.5. If necessary, extend the clearing time to 48–72 h. In this case, we recommend exchanging the clearing solution every 24 h with a freshly prepared Fructose-Glycerol-Clearing solution.

### Mounting optically cleared samples for confocal imaging

**Timing: ∼10 min**

26. Choose the iSpacer of the desired thickness (depending on the thickness of the sample) and stick it on a glass microscope slide.
27. Place the sample in the middle in a drop of Fructose-Glycerol-Clearing solution.
  a. Carefully place a coverslip on the top, avoiding the formation and trapping of bubbles.
  b. Use the DAPI channel to visualize and orient the sample (see troubleshooting section, problem 6).

**CRITICAL:** Mounting samples require some training -use optically cleared test tissue samples to train.

28. Let the samples settle for a few hours.
  a. Image the samples using a confocal microscope with the Z-stack scanning function for 3D imaging (see troubleshooting, problem 7).

**Figure 2:**
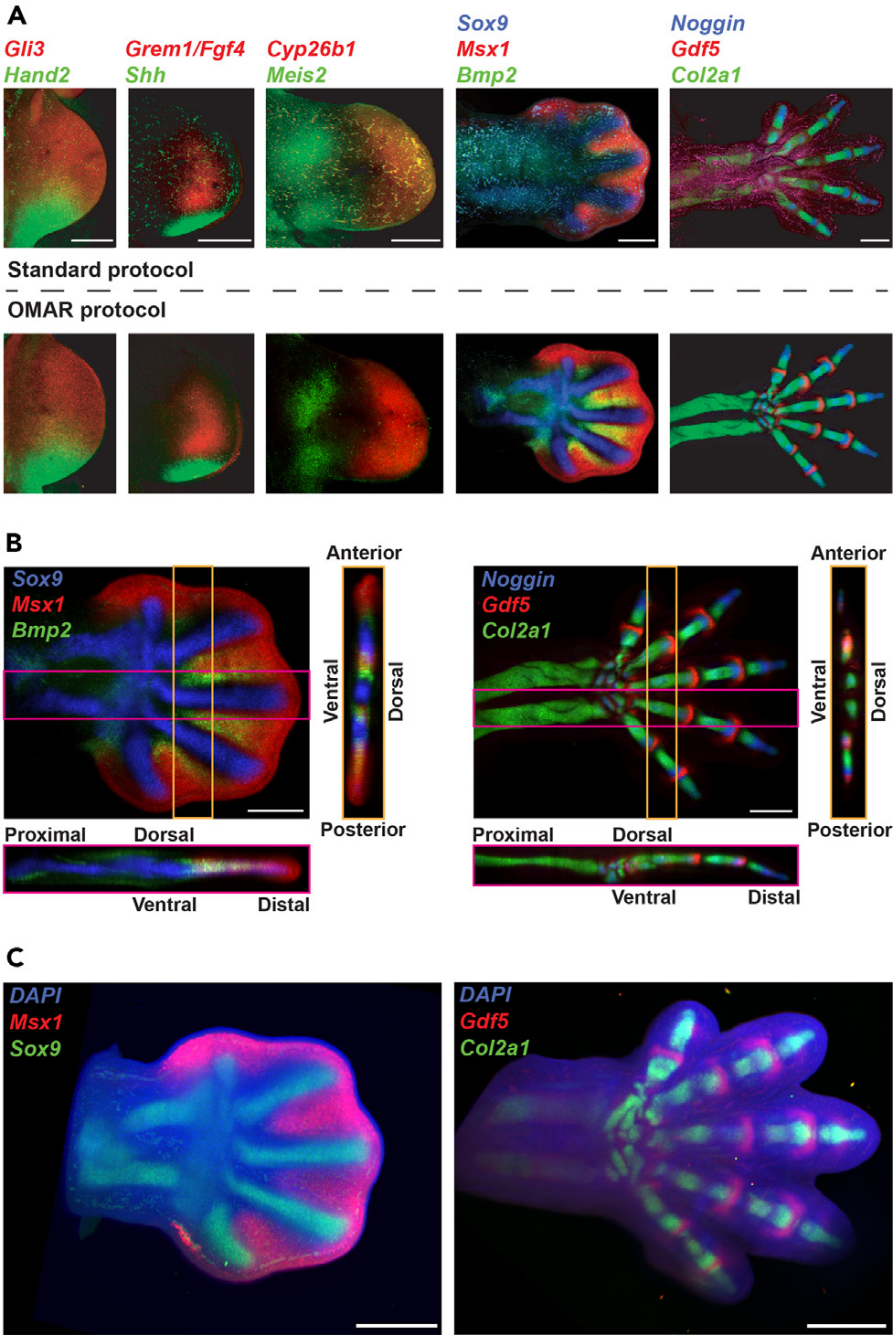
High signal-to-noise ratio and sensitive detection of multiple spatially distinct gene expression patterns in limb buds of different developmental stages (A–C) All limb buds shown in (A and B) are processed for and imaged by confocal microscopy without subsequent digital autofluorescence subtraction or masking. (A) Blood cell and blood vessel autofluorescence and probe penetration problems increase progressively with the more advanced stages of mouse limb bud development using a standard HCR RNA-FISH protocol (upper panels). In contrast, OMAR photobleaching in combination with SDS-detergent permeabilization reduces tissue autofluorescence improves probe penetration and signal-to-noise ratio at all stages analyzed (lower panels). (B) 3D reconstruction of the limb buds shown in the two rightmost images (lower panels in A) reveals the tissue integrity, even hybridization/signal detection, and very high signal-to-noise ratio throughout the limb buds. Scale bars: 300 μm. (C) Limb buds first analyzed by confocal microscopy (panels A and C) were remounted and imaged by light-sheet microscopy. Left panel: 3D volume rendering of a forelimb bud to detect both *Msx1* (red channel) and *Sox9* (green channel). Right panel: 3D volume rendering of a forelimb bud to detect *Gdf5* (red) and *Col2a1* (green). Nuclei are counterstained by DAPI (blue channel). Methods video S1 and S2 are available online for panel C. Scale bars: 500 μm.

**Pause point:** If required, store the mounted samples at 4°C in the dark for short-term storage (1–3 weeks) and at −20°C for long-term storage.

***Note:*** Using a spinning disk confocal microscope with a 10× objective (Visitron Spinning Disk Confocal) works well for acquiring Z-stacks of mouse limb buds up to E13.5 (dorsal to ventral direction) with appropriate resolution. For 2-dimensional maximum projection imaging, a step size of 5 μm is sufficient. Such 5 μm Z-stacks are adequate to reconstruct 3D images. However, stack sizes between 0.7-1 μm Z-spacing are recommended for high-resolution analysis with close to single-cell resolution. Representative 2-dimensional maximum projection results are shown in Figures 1D, 2A, and 2B.

### Agarose embedding and refractive index matching for light sheet imaging

**Timing: ∼24 h**

Prepare Fructose-Glycerol-Cleared samples (step 25) for light sheet microscopy as follows.

29. Prepare 1% or 2% agarose by dissolving low-melting-point agarose in 10 mM Tris pH 7.4.
  a. Let it cool to 40°C on a heat block with a magnetic stirrer.
30. Let the melted agarose stand in a water bath at 40°C until the microbubbles disappear from the agarose (check by eye).
31. Gently pour the agarose into a Tissue-Tek cryomold and let it cool.
  a. Transfer a limb bud sample using a plastic pipette.
  b. Orient the sample using a pipet tip.

***Note:*** Low-melting-point agarose takes approximately 5 min to solidify, which leaves sufficient time to orient the sample with a pipet tip.

32. Trim the excess agarose using a razor blade once it solidifies.
  a. Transfer the small cube-shaped sample block to a 2 mL microcentrifuge tube.

***Note:*** The refractive index of the Fructose-Glycerol-Clearing solution is 1.47.

33. Adjust the refractive index of the Fructose-Glycerol-Clearing solution to 1.45 by adding 10 mM Tris (pH 7.4).
  a. Add 1 mL of the Fructose-Glycerol-Clearing solution (refractive index 1.45) to the agarose-embedded sample tube.
  b. Gently shake on a rocking platform (20 oscillations/min) for 24 h at room temperature (24°C) in the dark.

**CRITICAL:** Measure the refractive index using a refractometer always before use. 24 h are sufficient to match the refractive index. If necessary, continue for an additional 24–48 h at 4°C in the dark (see troubleshooting, problem 7).

***Note:*** We use a Zeiss Lightsheet 7 microscope with the ZEN black 3.1 LS (version 9.3.10.393) software and lasers at a fixed wavelength of 561 nm and 638 nm. Dual side illumination is performed using the illumination air lenses LSFM foc 5×/0.1 and adjusted for a refractive index of 1.45. Fluorescence is detected using an EC Plan-Neofluar 5×/0.16 air detection objective, with the correction collar adjusted to the refractive index. Agarose-embedded samples are adhered to a metal plunger and mounted onto the sample holder. Then, the samples are immersed into a 5× clearing chamber filled with 30–40 mL of the refractive index-matched Fructose-Glycerol-Clearing solution (step 33). Manual alignment of the light sheet is performed using the 561 nm laser and adjusting the collars to the refractive index. The Z-stacks are acquired using the optimal step size of 3.77 μm to achieve the recommended Nyquist sampling. All acquired images are aligned and fused with Fiji and custom scripts using the BigStitcher plugin. The results are then converted to an IMARIS file that allows the reconstruction of the 3D volumes. Representative light sheet microscopy results are shown in Figure 2C and the Methods video S1 and S2.

Methods video S1. Light sheet 3D volume movie of a forelimb bud E12.5, related to step 33 The expression of *Msx1* (red) and *Sox9* (green). Nuclei are stained with DAPI (blue).

Methods video S2. Light sheet imaging on E13.5 forelimb buds, related to step 33 A 3D volume movie of a forelimb bud (E13.5) showing the *Gdf5* (red) and *Col2a1* (green) expression patterns. Nuclei are stained with DAPI (blue).

### Whole-mount immunofluorescence analysis

**Timing: ∼48 h**

A conventional immunohistochemistry protocol without antigen retrieval is used following fixation and photochemical bleaching (OMAR steps 2–10).

**CRITICAL:** Store the 4% PFA fixed embryos in PBS before dehydration for immunofluorescence.

**Figure 3:**
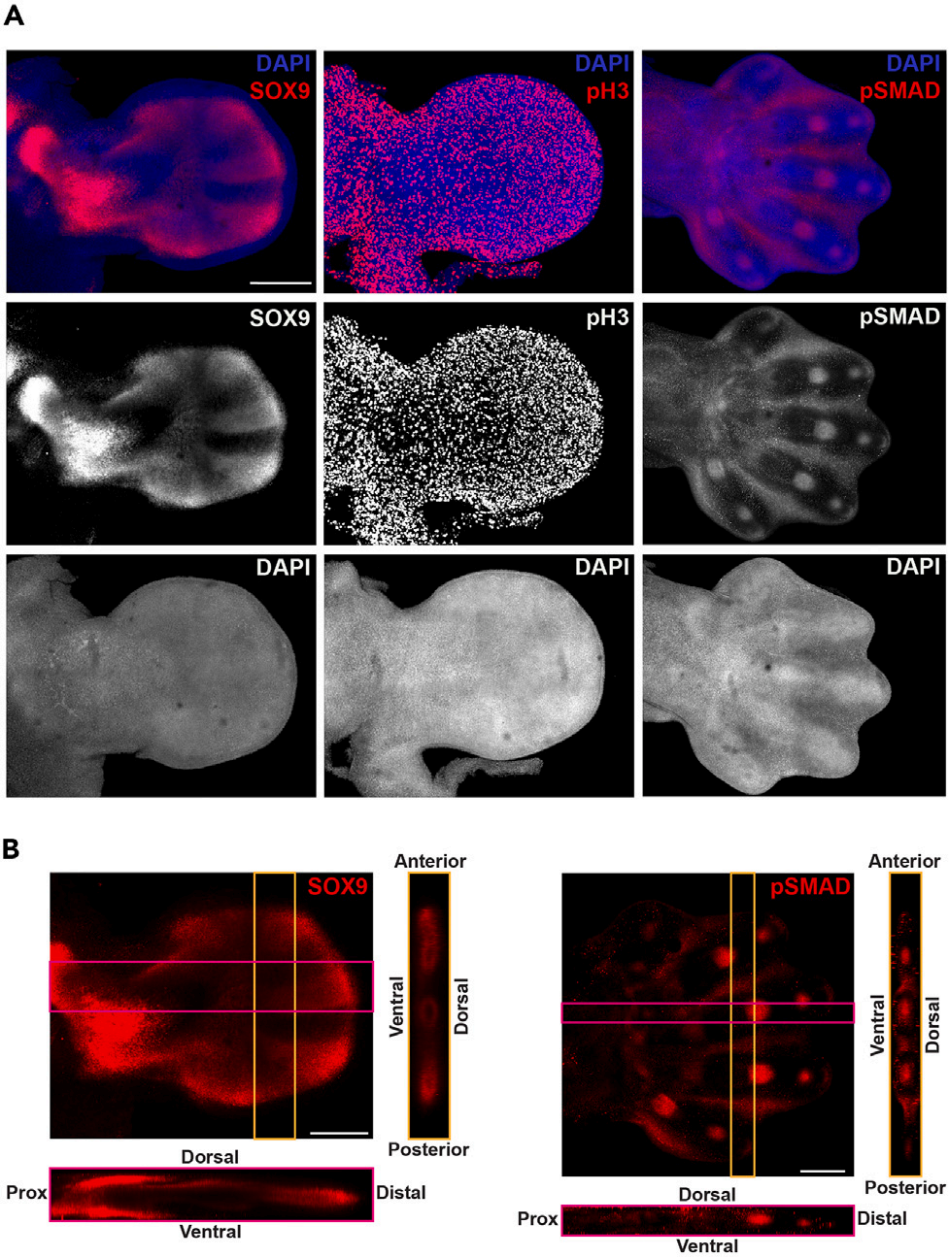
Whole-mount immunofluorescence staining following OMAR photobleaching (A) The spatial distribution of three proteins (red channel): SOX9 (left panels), phospho-Histone H3 (pH 3, middle panels), and phospho-SMAD1/5/9 (pSMAD, right panels). Nuclei are counterstained by DAPI (blue channel). (B) 3D reconstruction of the limb buds stained for SOX9 (left panels in A) and pSMAD (right panels in A) reveals the tissue integrity and detection of proteins throughout the limb bud with a very high signal-to-noise ratio. All limb buds shown are analyzed by confocal microscopy without digital post-processing apart from standard brightness and contrast adjustments. Scale bar: 500 μm.

As tissue autofluorescence issues often hamper whole-mount immunofluorescence analysis. We tested the OMAR protocol with several antibodies using mouse limb buds. While background fluorescence specific to antibodies is observed in some cases, high-quality results without the need for computational autofluorescence masking or subtraction are obtained with different antibodies (Figure 3). We recommend that the suitability of OMAR is established in a test experiment for all antibodies of interest (see troubleshooting section, problem 8).

***Note:*** Per 2 mL tube, 4–6 limb buds can be pooled. Steps 34–39 are carried out on a rocking platform (20 oscillations/min):

34. Permeabilize photochemically bleached samples 3 times for 10 min in 1 mL 0.4% Triton X-100 in PBS (at room temperature, 24°C).
  a. Remove 0.4% Triton X-100 in PBS. Pre-block the samples in 1 mL Blocking solution for at least 45 min (at room temperature, 24°C).
  b. Remove the Blocking solution. Incubate the samples with the primary antibody diluted in the Blocking solution for 16–20 h at 4°C.
  c. Remove the primary antibody solution. Wash the samples with 1 mL 0.1% Triton X-100 in PBS (pH 7.4) 6 times 30 min at room temperature (24°C).

***Note:*** The blocking solution stock is made without donkey serum and stored at 4°C for 6 months. Fresh donkey serum is added to an aliquot just before use.

***Note:*** The optimal primary antibody dilution has to be determined for each antibody by a titration test. In general, primary antibody dilutions in the range of 1:50-1:200 are recommended as starting points.

If necessary, the primary antibody incubation can be extended for 48–72 h.

**CRITICAL:** Centrifuge the diluted primary antibody at 1500 g in a tabletop microfuge for 10 min and immediately transfer the supernatant to a fresh tube to remove any precipitates.

35. Transfer the samples to a new microcentrifuge tube.
  a. Add the secondary antibody in 0.4% Triton X-100 in PBS, 1% BSA, and 10% serum that contains DAPI (1:1000 dilution).
  b. Incubate for 16–20 h at 4°C in the dark.

***Note:*** In general, secondary antibody dilutions in the range of 1:200-1:2000 are recommended.

36. Remove the secondary antibody solution.
  a. Wash the samples with 1 mL 0.1% Triton X-100 in PBS (pH 7.4) for 3 h at room temperature (24°C).
  b. Change the solution minimally 6 times, i.e., every 30 min.

***Note:*** The samples are now ready for optical clearing (continuing from step 25 of the core protocol). Representative results using some antibodies that work well using this whole-mount protocol are shown in Figure 3.

## Expected outcomes

To reliably eliminate autofluorescence, we developed a streamlined protocol that combines OMAR (Figures 1A–1C) with SDS detergent-based tissue permeabilization to achieve a superior signal-to-noise ratio for whole-mount HCR RNA-FISH analysis (Figures 1D and 2). For all limb bud stages, but in particular, for developmentally advanced stages (E11.0-13.5), OMAR drastically reduces autofluorescence in comparison to untreated samples (Figure 2A). This results in superior spatial resolution of graded RNA distributions in limb buds and developing skeletal elements (Figure 2A). In fact, 3D Z-stack reconstructions of developmentally advanced limb buds (E12.5; 13.5) shows that the tissue integrity is not significantly altered by photochemical bleaching. Furthermore, the SDS detergent-based permeabilization results in homogeneous penetration and hybridization of HCR probes (dorsal-ventral and proximal-distal axes; Figure 2B). In addition, this method, in combination with water-based sample clearing, allows the acquisition of high-quality 3D and 2D images using light sheet microscopy (Figures 2C, Methods video S1, and S2). Another asset of this protocol is that it also works with 4% PFA fixed and dehydrated samples stored for over a year without any notable decrease in signal-to-noise ratios. In addition, we establish that OMAR can be used for whole-mount immunohistochemistry, yielding high-quality results with different antibodies (Figure 3). Finally, autofluorescence reduction is crucial for the acquisition of high-quality raw images not requiring digital post-processing such as masking tissue autofluorescence. The reproducibility and efficiency of OMAR indicate that it will work with other embryonic tissues and vertebrate embryos.

## Limitations

The OMAR protocol described is tested on mouse embryos fixed in 4% PFA for 12–16 h. Here, we discuss limitations and possible solutions: Firstly, the potential use of other fixatives needs to be tested as autofluorescence, and tissue integrity depends on the fixative strength and the de- and rehydration procedure. Also, alternative long-term storage conditions such as 100% Ethanol (for whole-mount HCR RNA-FISH) or 80% Methanol and 20% DMSO (for whole-mount immunofluorescence) need to be tested first (see steps 2–4). Secondly, his protocol is tested on mouse embryonic tissue with a thickness ranging from 50 microns to 450 microns. It will likely work for other embryonic tissues and embryos from different species. However, whether epithelial structures such as the early developing heart and neuroepithelia withstand the OMAR photochemical bleaching needs to be established (see steps 7–10). Assess autofluorescence removal versus tissue integrity in a pilot time course experiment such as bleaching of samples two or three times 15 min, two times 30 min and/or two times 45 min. If necessary, the H_2_O_2_ and NaOH concentrations can be reduced to 3% and 10 mM, respectively. Finally, OMAR was not tested on embryos older than E14.5, postnatal and adult tissues and organs. We would expect that increasing the number of photochemical bleaching cycles is required for autofluorescence reduction and extending detergent-based permeabilization to 3–4 h for developmentally advanced and postnatal stages.

## Troubleshooting

### Problem 1

No bubbles formed during OMAR photochemical bleaching.

### Potential solution

• The problem could arise due to insufficient lighting or loss of H_2_O_2_ activity.
• Please check that the lights are turned on high power. We recommend the use of a high-power cold white LED light source.
• Replace the H_2_O_2_ with a fresh stock bottle stored at 4°C following the manufacturer’s instructions.
• Always prepare the bleaching solution immediately before use (for every photobleaching cycle).

### Problem 2

Tissue samples stick to the plastic of the multi-well plate during photochemical bleaching.

### Potential solution

• Add PBS to the wells in question, wait 3–5 min, and gently flush using a plastic pipette until the sample detaches. Do not use forceps.

### Problem 3

Autofluorescence is not reduced or eliminated after two rounds of photochemical bleaching.

### Potential solution

• In our hands, 2 × 30 min or 2 × 45 min works well for limb buds of different stages. However, depending on sample size, thickness, and tissue density, the photochemical bleaching time and number of cycles need to be empirically determined in a pilot experiment (see steps 7–10).
• We recommend increasing the number of photobleaching cycles. A pilot test 2–3 × 15 min, 2 × 30 min, 2 × 45 min and 4 × 30 min should be done.
• In some HCRs, we noted that the detection of low-expressed genes is paralleled by enhanced levels of background autofluorescence, resulting in reduced signal-to-noise ratios. For genes expressed at low levels or in a graded manner, we recommend using far-red spectrum fluorophores (e.g., 647).
• We noted that the high-power spotlight LEDs (Figure 1A) perform better than light pads (Figure 1B). Therefore, we recommend using high-power spotlight LEDs for developmentally advanced stages.

### Problem 4

Bubbles trapped in the tissue samples during photochemical bleaching may cause tissue damage.

### Potential solution

• A common problem is incomplete tissue dehydration (after fixation) or rehydration (before photobleaching). We recommend increasing the times for all steps of the de-/re-hydration series and leaving the samples for 12–24 h in 100% Methanol with additional changes.
• Optimize the photochemical bleaching time and number of cycles for the tissue of interest in a pilot experiment (see problem 3).
• Perform the washing steps after photobleaching in a shaker at 300 rpm. Wash longer in PBST, as this will aid in removing the trapped bubbles.
• If the problem persists, consider reducing the H_2_O_2_ and NaOH concentrations to 3% and 10 mM, respectively. However, lowering the oxidizing agent concentration will likely hamper tissue autofluorescence suppression.

### Problem 5

Failure to detect the expression of the gene of interest by HCR RNA-FISH.

### Potential solution

• We have noted that some probes only work with a specific fluorophore/color.
• We recommend using fluorophores 488, 514, or 594 for genes expressed at high levels in combination with far-red fluorophores (e.g., 647) for genes expressed in a graded manner or at low levels.

### Problem 6

Inclusion or floating fluorescent contaminations and/or dust in cleared and mounted samples.

### Potential solution

• Unmount the sample, wash it in freshly prepared Fructose-Glycerol-Clearing solution for a couple of hours, then carefully remount.
• Always use a clean and dust-free place for mounting and embedding samples.

### Problem 7

Blurry or striped confocal or light-sheet images.

### Potential solution

• This is likely due to insufficient clearing and a heterogeneous refractive index within the tissue.
• Initially, do not unmount the sample; store it in the dark at 4°C for a couple of days and repeat the imaging.
• If that does not solve the problem, unmount the sample and clear it longer in a freshly prepared Fructose-Glycerol-Clearing solution.
• For light-sheet imaging, check that the refractive index of the Fructose-Glycerol-Clearing solution is perfectly matched (steps 29–33). Consider increasing refractive index matching time.

### Problem 8

This protocol does not work with particular antibodies.

### Potential solution

• Consider reducing the fixation time to 2–4 h instead of overnight.
• For a fraction of antibodies tested (not shown), background fluorescence is detected due to stickiness or tapping of the primary antibody.
• Some antibodies might not work with the protocol described above. They may require an antigen retrieval step before photobleaching.^13^
• In these cases, the use of other protocols is recommended.^14^^,^^15^

## Resource availability

### Lead contact

Further information and requests for resources and reagents should be directed to and will be fulfilled by the lead contact, Rushikesh Sheth (rushikesh.sheth@unibas.ch).

### Materials availability

All material is freely available upon request.

## Data Availability

The protocol includes all analyzed data shown in Figures 1, 2, and 3.
